# Prognostic Value of Overexpressed p16^INK4a^ in Vulvar Cancer: A Meta-Analysis

**DOI:** 10.1371/journal.pone.0152459

**Published:** 2016-03-31

**Authors:** Hanyu Cao, Si Wang, Zhenyu Zhang, Jiangyan Lou

**Affiliations:** 1 Department of Gynecology and Obstetrics, West China Second University Hospital, Sichuan University, Chengdu City, Sichuan Province, China; 2 Genome Stability Laboratory, West China Second University Hospital, Sichuan University, Chengdu City, Sichuan Province, China; 3 Key Laboratory of Birth and Related Diseases of Women and Children, Sichuan University Ministry of Education, Chengdu City, Sichuan Province, China; Rudjer Boskovic Institute, CROATIA

## Abstract

**Objective:**

This study aimed to examine the prognostic value of overexpressed p16^INK4a^ in vulvar cancer. Although the tumor suppressor p16^INK4a^ has been shown to be of prognostic value in a wide variety of cancers and precancerous lesions, its role in the vulvar cancer is still unclear.

**Methods:**

All publications in English language on the association between p16^INK4a^ and clinicopathological features of vulvar cancer were searched from Pubmed, Embase, and Web of Science, and those in Chinese language were identified manually and online from the China National Knowledge Infrastructure. Strict inclusion and exclusion criteria were followed. Odds ratios(ORs) or risk ratios(RRs) with 95% confidence intervals(CIs) were pooled to assess the strength of association. Publication bias was estimated using funnel plots and the Egger’s regression test.

**Results:**

A total of 17 studies with 2309 patients were included. The p16^INK4a^ overexpression was found to correlate significantly with the lower International Federation of Gynecology and Obstetrics stage(I+II vs III+IV; OR = 0.60,95%CI:0.41–0.86,*P* = 0.006),negative lymph node metastasis(negative vs positive; OR = 0.61,95%CI:0.39–0.95,*P* = 0.029),patient’s age<55(OR = 0.54,95%CI:0.31–0.96,*P* = 0.034),human papillomavirus–positive status(OR = 0.01,95%CI:0.00–0.11,*P*<0.001),and higher overall survival(RR = 0.53,95%CI = 0.35–0.80,*P* = 0.003).

**Conclusion:**

The p16^INK4a^ might be associated with a higher survival and indicates better prognosis of vulvar cancer.

## Introduction

Vulvar cancer is the fourth most common malignancy in the female genital tract, accounting for 5% of all gynecological cancers[1].With an estimate of over 5000 new cases and over 1000 deaths in the United States each year, the incidence of vulvar cancer keeps on rising, particularly in young women [1,2].The most frequent histological type of vulvar cancer is squamous cell carcinoma(SCC), which represents 80%–90% of the cases and can be divided into two groups based on whether they are related to human papillomavirus (HPV) infection[3,4]. One is the HPV-associated type including the warty or basaloid SCC, and the other is the HPV-independent keratinizing SCC[5,6].Radical surgery is the main modality of treatment for vulvar cancer, sometimes in combination with presurgical or postsurgical radiotherapy or chemotherapy depending on the tumor size and invasion, but often causes overtreatment in early stage cases when patients present nonmetastasis of lymph node[7]. For this reason, tumor biological markers are needed to predict clinical behavior and metastatic potential of vulvar cancer.

The p16^INK4a^ protein is a cyclin-dependent kinase inhibitor that can act on cyclin-dependent kinases(CDKs) and thus inactivate the cell cycle[8].The loss of p16ink4a expression seems to be an early event in carcinogenesis. Over the past decades, the prognostic value of p16^INK4a^ protein has been evaluated in a wide variety of cancers [9–14].Among these, the study of Hellman et al[15] concluded that p16^INK4A^ expression might be used as a marker for HPV positivity in vaginal carcinoma, which shares a very similar etiology with vulvar cancer. However, its role and the etiological effect of its combination with the HPV status in vulvar cancer remain somehow unclear. Tringler and Dong reported a significant association between p16^INK4a^ and longer survival of vulvar cancer patients, while Trietsch denied its independent prognostic role[16–18].In this regard, the present study performed a meta-analysis to explore the prognostic value of the overexpressed p16INK4a in vulvar cancer.

## Methods

### Search strategy

To identify all articles that investigated the association of p16^INK4a^ expression and vulvar cancer, a literature search of PubMed database, Embase, Web of Science, and China National Knowledge Infrastructure was conducted between January 1991 and August 2015. All relevant articles were retrieved using the following search terms: “p16 or p16^INK4a^” and “vulvar neoplasm or vulvar neoplasms”. References of the retrieved publications were also screened for other relevant studies. For multiple publications from the same population, only the largest-scale study was included. Study selection was achieved by two investigators independently, according to the inclusion and exclusion criteria, by screening the title, abstract, and full-text. Any dispute was solved by discussion. The language of publication was restricted to English and Chinese. Only research articles were included. If an article reported results including different studies, each study was treated as a separate comparison in the present meta-analysis.

### Inclusion criteria

The following inclusion criteria were followed in selecting studies for the current meta-analysis: Studies in which the diagnosis of vulvar cancer was proven by histopathological methods; studies of p16^INK4a^ expression that were based on vulvar cancer tissue (after either surgical excision or biopsy sampling);specimens examined by immunohistochemistry; and all studies on the correlation of p16^INK4a^ expression with clinicopathological markers or with the HPV status and the association of p16^INK4a^ overexpression on the overall survival (OS) or disease-free survival (DFS) or recurrence of vulvar cancer patients. For inclusion into the analysis, no limitation was set on the minimum number of patients of each study. In the case of multiple articles by the same group based on similar patients and using same detection methods, only the largest or the most recent article was included.

### Exclusion criteria

The exclusion criteria were defined as follows: Studies based on serum or any other kinds of specimen, and studies using methods other than immunohistochemistry to examine specimens.

### Data extraction

Three investigators (Hanyu Cao, Si Wang, and Zhenyu Zhang) extracted independently all articles complying with the aforementioned inclusion criteria. Any discrepancy was resolved by discussion until an agreement was reached between the investigators. The following information was collected from each publication: the first author’s name, publication year, patient’s country, tumor stage, technique, percentage of p16^INK4a^ positive cells, number of patients, cutoff value of overexpression of p16^INK4a^ in cases and controls.

### Statistical analysis

Odds ratios(OR) and 95% confidence intervals (CIs) were pooled to evaluate the association between p16^INK4a^ expression and clinical or histopathological features of vulvar cancer including histological grade, International Federation of Gynecology and Obstetrics (FIGO) stage, lymph node metastasis, age, and HPV status. Risk ratios(RRs) and 95% CIs were synthesized to determine the correlation between p16^INK4a^ expression and 5-year OS. Heterogeneity was examined with *I*^2^ statistics interpreted as the proportion of total variation contributed by between-study variations. Heterogeneity was investigated by the Cochran’s chi-square Q test with a significance level of *P*<0.10 and *I*^2^>50%.In this case, the random-effects model was used to estimate the pooled ORs [19].Otherwise, the pooled ORs were estimated by the fixed-effects model. RRs were pooled to evaluate the association of p16^INK4a^ expression and survival outcome data. The Engauge Digitizer Version 4.1 software (free software downloaded from http://sourceforge.net) was used when the survival data could not be extracted directly according to previous studies[20]. Begg’s funnel plots and the Egger’s linear regression test were performed to investigate publication bias [21]. All statistical tests were performed with the stata 12.0 software(Stata Corp, TX, USA).

## Results

### Study inclusion and characteristics

**Fig 1:**
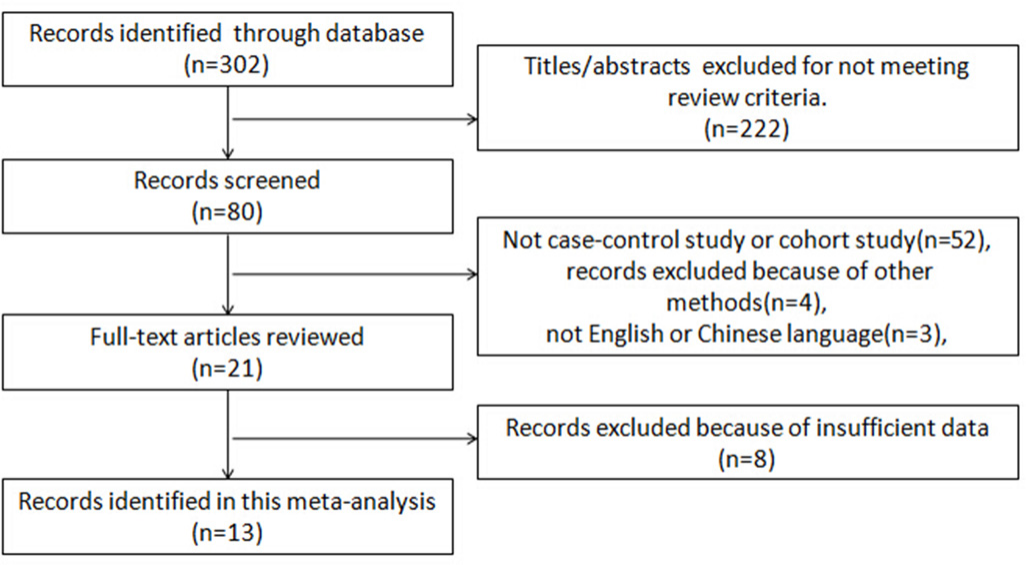
Flow chart demonstrating studies processed for inclusion in the meta-analysis.

As shown in Fig 1, a total of 17 eligible studies comprising 2309 patients from different countries were included in this meta-analysis, with the number of patients ranging from 4 to 1287 per study[16–17,22–36].Among them, eight studies dealt with the FIGO stage, and six studies presented data on lymph node metastasis. Three studies presented data classified according to patients’ age (55 years), and seven studies reported histological grade. In addition, four studies reported the association between p16^INK4a^ and the HPV status, whereas only one study[34] included the DFS and OS data concerning the HPV status. Moreover, only three studies provided sufficient data on OS related to p16^INK4a^ expression. All of them used the immunohistochemistry method with different cut-off values. The main characteristics of these studies are summarized in Table 1.

**Table 1:** Main Characteristics of all Eligible Studies.

Study	Year	Patient's country	Tumor stage	Technique	Percentage of p16INK4a positive cells (%)	Number of patients	Cutoff(IHC)
Chan MK	1998	China	FIGO	IHC	72	30	ND
Zheng	2000	China	FIGO	IHC	40.38	52	ND
Ma	2002	China	FIGO	IHC	64	25	Staining grade>2
Hao	2004	China	ND	IHC	40	30	ND
Knopp S	2004	Norway	FIGO	IHC	31	224	5%
Mauricio	2006	Brazil	ND	IHC	24.3	37	5%
Santos M	2006	Spain	ND	IHC	18	92	25%
van der Avoort	2006	The Netherlands	ND	IHC	75	16	25%
Tringler B	2007	Austria	FIGO	IHC	43	80	ND
Wei	2008	China	FIGO	IHC	29	21	ND
Alonso I	2011	Spain	FIGO	IHC	20	98	Diffuse staining
Guerrero D	2011	Spain	TNM	IHC	70	30	1%
de Sanjosé S	2013	39 Countries	ND	IHC	29	1287	ND
Ma HM	2013	China	FIGO	IHC	90.3	36	Staining grade>2
Lavorato-Rocha	2013	Brazil	FIGO	IHC	29	139	5%
Missaoui N	2014	Tunisia	FIGO	IHC	73	15	ND
Dong	2015	America	ND	IHC	52	97	70%

FIGO, International Federation of Gynecology and Obstetrics; IHC, immunohistochemistry; ND, no data; TNM, tumor node metastases.

### Correlation of p16^INK4a^ expression with clinicopathological parameters

**Fig 2:**
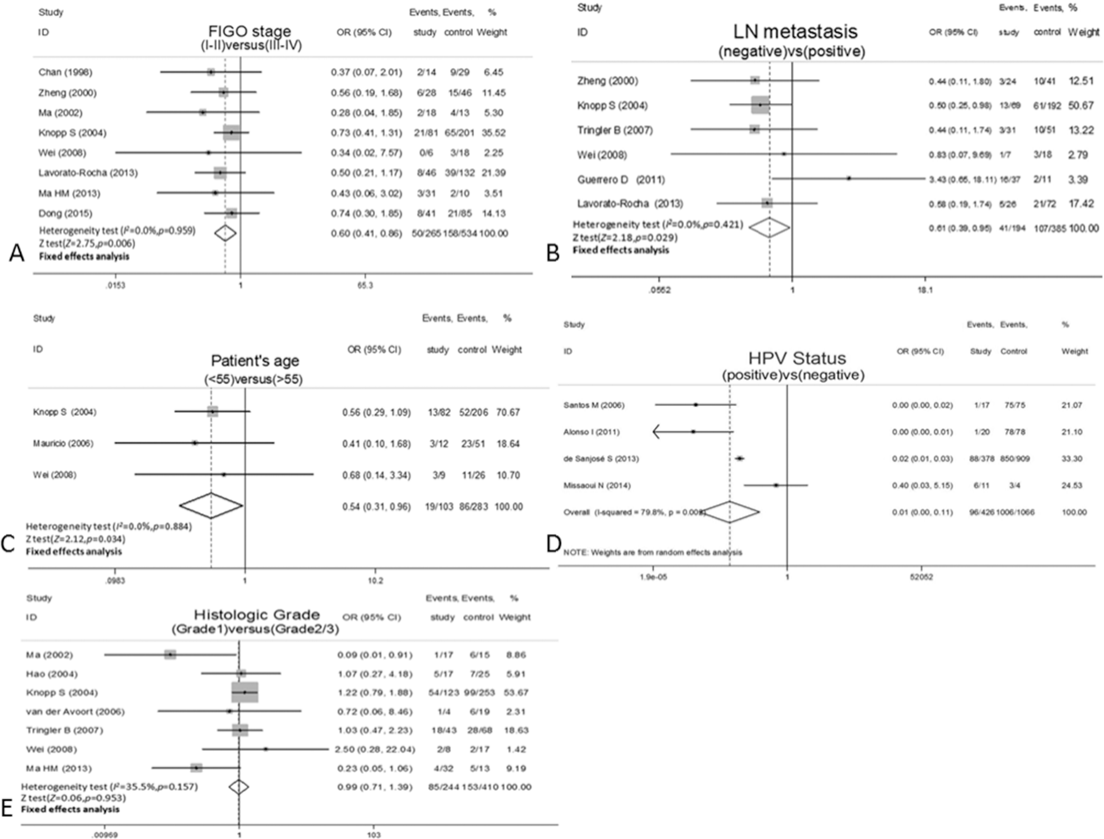
**Forest plot for the association between p16INK4a expression and clinicopathological markers**(A) FIGO stage.(B) LN metastasis. (C) Patient's age. (D) HPV status. (E) Histological grade. FIGO, International Federation of Gynecology and Obstetrics; LN, lymph node; HPV, human papillomavirus.

As shown in Fig 2, the overexpression of p16^INK4a^ was significantly associated with the lower FIGO stage of I–II(OR = 0.60,95%CI:0.41–0.86,*P* = 0.006,fixed effect; Fig 2A), no lymph node metastasis(OR = 0.61,95%CI:0.39–0.95,*P* = 0.029,fixed effect; Fig 2B), patient’s age<55(OR = 0.54,95%CI:0.31–0.96,*P* = 0.034,fixed effect; Fig 2C), and HPV-positive status(OR = 0.01,95%CI:0.00–0.11,*P*<0.001,random effect; Fig 2D).However, the overexpression of p16^INK4a^ was not correlated with the histological grade(OR = 0.99,95%CI:0.71–1.39,*P* = 0.953,fixed effect; Fig 2E).

### Correlation of p16^INK4a^ expression with OS

**Fig 3:**
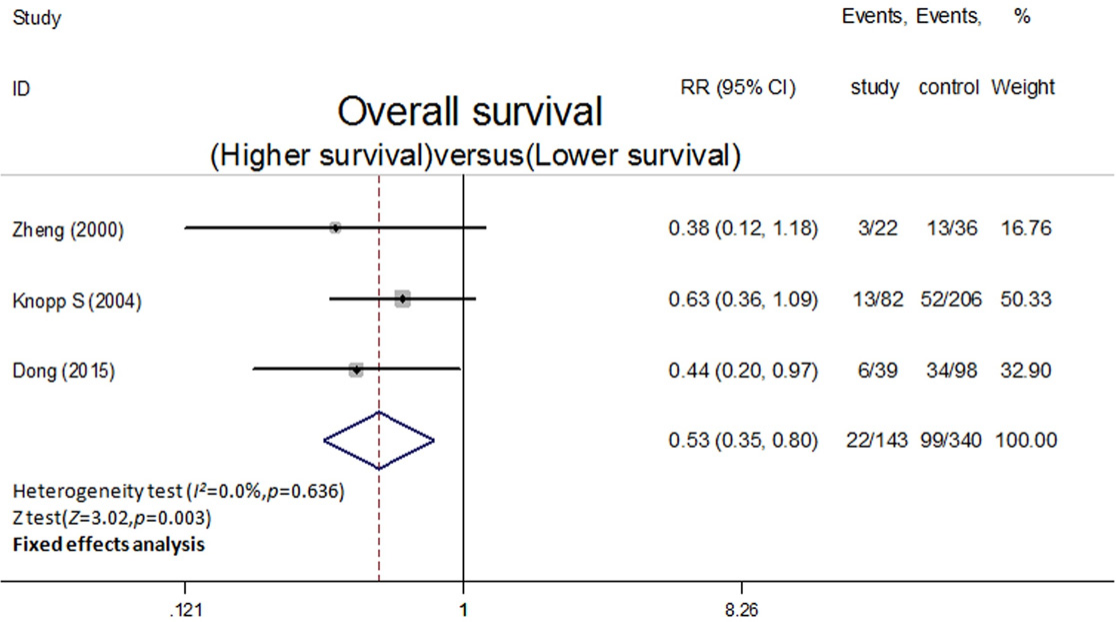
Forest plot for the association between p16INK4a expression and OS.

Five-year OS rates were extracted from three studies and were pooled in this meta-analysis using the method mentioned earlier. The overexpression of p16^INK4a^ was significantly correlated with a higher OS(RR = 0.53,95%CI = 0.35,0.80,*P* = 0.003,fixed effect; Fig 3) with no heterogeneity(*I*^2^ = 0.0%,*P* = 0.636).

### Publication Bias

**Fig 4:**
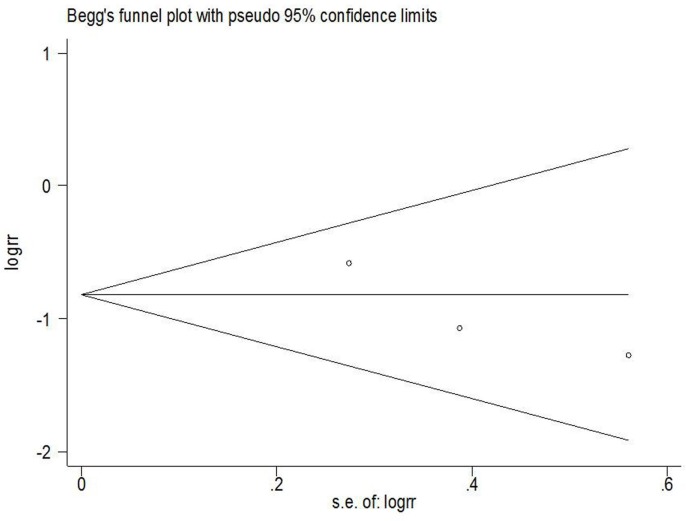
A funnel plot analysis on the detection of publication bias in the meta-analysis of the prognostic value of p16ink4a in the OS of vulvar cancer.

Begg’s funnel plots and Egger’s test were used to assess whether publication bias existed in the 5-year OS studies. No obvious evidence of asymmetry was found in the funnel plot of OS(*P* = 0.217; Fig 4).

## Discussion

P16^INK4a^,as a member of the INK4a family, is known to inactivate cell cycle progression and differentiation by interfering with cyclin D–CDK4/6 assembly and thus block the G1 to S transition[37].The functional loss of INK4a family has been found in variable human cancers. Some previous studies have provided evidences indicating that p16^INK4a^ is a potential tumor suppressor whose overexpression can inhibit abnormal cell growth, whereas downregulation may lead to tumorigenesis[38]. Although p16^INK4a^ has been shown to be of prognostic value in a wide variety of cancers and precancerous lesions such as gastrointestinal stromal tumor, non-small lung cancer, osteosarcoma, cervical cancer, and cervical intraepithelial neoplasia, evidence of correlation with vulvar cancer is lacking[12–13,39–41].

Vulvar cancer is uncommon but represents a certain group in the carcinoma of lower genital tract in female. As the treatment for vulvar cancer tends to be more conservative and individualized over the past decade, acquaintance with the prognostic factors seems more essential and is of great value for early diagnosis, but is still a challenge because of scanty evidence compared with other gynecological malignancies[42].It was found that FIGO stage, lymph node metastasis, and histological grade are the most important prognostic factors in vulvar cancer[43–45]. The age of patients is also a predominant and independent factor in the 5-year survival of vulvar cancer[46]. This study is perhaps the first meta-analysis that evaluated the prognostic value of p16^INK4a^ in vulvar cancer.

The present meta-analysis using pooled ORs demonstrated that p16^INK4a^ overexpression correlates significantly with the lower FIGO stage, negative lymph node metastasis, and younger age, consistent with its well-known function of suppressing tumor invasion[47–49].The pooled RR of OS reveals that p16^INK4a^ overexpression indicates a better prognosis for patients diagnosed with vulvar cancer. The results indicate that p16^INK4a^ may be a potential biomarker for the diagnosis and prognosis of vulvar cancer. Although evidence shows improved survival of HPV-induced and p16-positive carcinoma associated with head and neck cancer[50], no detailed studies are found presenting the survival data including both HPV status and p16^INK4a^.A very few studies presented recurrence rate related to p16 concerning vulvar squamous cell carcinoma (VSCC) after repeated searches for several times, which may be due to the low frequency of VSCC. Among the included studies, HPV-positive rate independent of p16^INK4a^ ranges between 0% and 41% [23,25,27,34],which is in accordance with the fact that vulvar cancer presents an overall HPV infection rate of only 30%–40%[51–52].Moreover, the analysis outcome shows that p16^ink4a^ overexpression correlates significantly with the HPV-positive status, strongly supporting the etiological role of HPV infection in the development of vulvar cancer. The E7 and E6 oncoproteins encoded by HPV can be integrated into the host genome, binding to the tumor suppressor proteins pRB and p53, respectively, and leading to their deregulation, which results in the overexpression of p16^INK4A^ as a means of genetic instability control[53].All these may reasonably lead to the speculation that p16INK4a is only associated with HPV-induced vulvar carcinoma.

However, this study has some limitations that should be taken into consideration. First, this study included a small sample size. A total of 817 patients may not be able to provide statistical evidence convincing enough to prove the role of p16^INK4a^ in vulvar cancer prognosis. In addition, studies concerning the survival rate of vulvar cancer are even less. Second, the arbitrarily selected cutoff value of positive p16^INK4a^ expression varies in different studies. This may be caused by the lack of a standardized immunostaining point decided by a combination of intensity and the variable methodological factors in immunohistochemistry such as storage time, fixation method, and different antibodies[54], which may result in an inaccurate outcome. All studies were evaluated based on a review of the entire histological section, while some declared the evaluation of the immunostaining results by one or two pathologists at the same time according to previous experience and established criteria in vulvar neoplasia or for uterine cervix[55,56],which ensure their reliability. Third, treatment methods for each patient differed to a certain extent for their multiple kinds of clinical and histological features, which should not be ignored. For these reasons, further research with a larger sample size and more evidence are needed to confirm the findings of this study.

In conclusion, despite the limitations, the results of the present meta-analysis suggested that p16^INK4a^ overexpression was associated with a favorable prognosis in patients with vulvar cancer. The assessment of p16^INK4a^ expression is capable of providing better prognostic information for patients with vulvar cancer.

## Supporting Information

S1 PRISMA Checklist PRISMA 2009 Checklist. (DOC)

## References

[1] Siegel RL, Miller KD, Jemal A. Cancer statistics, 2015. CA: a cancer journal for clinicians. 2015;65(1):5–29. 10.3322/caac.21254 . 25559415

[2] Lanneau GS, Argenta PA, Lanneau MS, Riffenburgh RH, Gold MA, McMeekin DS, et al Vulvar cancer in young women: demographic features and outcome evaluation. American journal of obstetrics and gynecology. 2009;200(6):645e1-5. 10.1016/j.ajog.2009.01.014 . 19286150

[3] Crum CP. Carcinoma of the vulva: epidemiology and pathogenesis. Obstetrics and gynecology. 1992;79(3):448–54. . 1310806 10.1097/00006250-199203000-00025

[4] Kagie MJ, Kenter GG, Zomerdijk-Nooijen Y, Hermans J, Schuuring E, Timmers PJ, et al Human papillomavirus infection in squamous cell carcinoma of the vulva, in various synchronous epithelial changes and in normal vulvar skin. Gynecologic oncology. 1997;67(2):178–83. 10.1006/gyno.1997.4834 . 9367704

[5] Kurman RJ, Toki T, Schiffman MH. Basaloid and warty carcinomas of the vulva. Distinctive types of squamous cell carcinoma frequently associated with human papillomaviruses. The American journal of surgical pathology. 1993;17(2):133–45. . 8380681 10.1097/00000478-199302000-00005

[6] Sideri M, Jones RW, Wilkinson EJ, Preti M, Heller DS, Scurry J, et al Squamous vulvar intraepithelial neoplasia: 2004 modified terminology, ISSVD Vulvar Oncology Subcommittee. The Journal of reproductive medicine. 2005;50(11):807–10. . 16419625

[7] Scheistroen M, Nesland JM, Trope C. Have patients with early squamous carcinoma of the vulva been overtreated in the past? The Norwegian experience 1977–1991. European journal of gynaecological oncology. 2002;23(2):93–103. . 12013120

[8] Ruas M, Peters G. The p16INK4a/CDKN2A tumor suppressor and its relatives. Biochimica et biophysica acta. 1998;1378(2):F115–77. . 9823374 10.1016/s0304-419x(98)00017-1

[9] Chen YZ, Liu D, Zhao YX, Wang HT, Gao Y, Chen Y. Relationships between p16 gene promoter methylation and clinicopathologic features of colorectal cancer: a meta-analysis of 27 cohort studies. DNA and cell biology. 2014;33(10):729–38. 10.1089/dna.2013.2253 . 24979649

[10] Rocco JW, Sidransky D. p16(MTS-1/CDKN2/INK4a) in cancer progression. Experimental cell research. 2001;264(1):42–55. 10.1006/excr.2000.5149 . 11237522

[11] Honoki K, Stojanovski E, McEvoy M, Fujii H, Tsujiuchi T, Kido A, et al Prognostic significance of p16 INK4a alteration for Ewing sarcoma: a meta-analysis. Cancer. 2007;110(6):1351–60. 10.1002/cncr.22908 . 17661343

[12] Zong L, Chen P, Jiang J, Wang H, Wang L. Correlation between p16 expression and malignant risk of gastrointestinal stromal tumor: evidence from nine studies. Hepato-gastroenterology. 2012;59(117):1458–63. 10.5754/hge11473 . 22094995

[13] Lou-Qian Z, Rong Y, Ming L, Xin Y, Feng J, Lin X. The prognostic value of epigenetic silencing of p16 gene in NSCLC patients: a systematic review and meta-analysis. PloS one. 2013;8(1):e54970 10.1371/journal.pone.0054970 ; PubMed Central PMCID: PMCPmc3555860. 23372805 PMC3555860

[14] Jiang W, Wang PG, Zhan Y, Zhang D. Prognostic value of p16 promoter hypermethylation in colorectal cancer: a meta-analysis. Cancer investigation. 2014;32(2):43–52. 10.3109/07357907.2013.861476 . 24410593

[15] Hellman K, Lindquist D, Ranhem C, Wilander E, Andersson S. Human papillomavirus, p16(INK4A), and Ki-67 in relation to clinicopathological variables and survival in primary carcinoma of the vagina. British journal of cancer. 2014;110(6):1561–70. 10.1038/bjc.2014.32 ; PubMed Central PMCID: PMCPmc3960612. 24525695 PMC3960612

[16] Tringler B, Grimm C, Dudek G, Zeillinger R, Tempfer C, Speiser P, et al p16INK4a expression in invasive vulvar squamous cell carcinoma. Applied immunohistochemistry & molecular morphology: AIMM / official publication of the Society for Applied Immunohistochemistry. 2007;15(3):279–83. 10.1097/01.pai.0000213118.81343.32 . 17721272

[17] Dong F, Kojiro S, Borger DR, Growdon WB, Oliva E. Squamous Cell Carcinoma of the Vulva: A Subclassification of 97 Cases by Clinicopathologic, Immunohistochemical, and Molecular Features (p16, p53, and EGFR). The American journal of surgical pathology. 2015;39(8):1045–53. 10.1097/pas.0000000000000454 . 26171917

[18] Trietsch MD, Spaans VM, ter Haar NT, Osse EM, Peters AA, Gaarenstroom KN, et al CDKN2A(p16) and HRAS are frequently mutated in vulvar squamous cell carcinoma. Gynecologic oncology. 2014;135(1):149–55. 10.1016/j.ygyno.2014.07.094 . 25072932

[19] DerSimonian R, Kacker R. Random-effects model for meta-analysis of clinical trials: an update. Contemporary clinical trials. 2007;28(2):105–14. 10.1016/j.cct.2006.04.004 . 16807131

[20] Parmar MK, Torri V, Stewart L. Extracting summary statistics to perform meta-analyses of the published literature for survival endpoints. Statistics in medicine. 1998;17(24):2815–34. . 9921604 10.1002/(sici)1097-0258(19981230)17:24<2815::aid-sim110>3.0.co;2-8

[21] Egger M, Smith GD. Bias in location and selection of studies. BMJ (Clinical research ed). 1998;316(7124):61–6. ; PubMed Central PMCID: PMCPmc2665334. 9451274 10.1136/bmj.316.7124.61 PMC2665334

[22] Chan MK, Cheung TH, Chung TK, Bao SY, Zhao CL, Nobori T, et al Expression of p16INK4 and retinoblastoma protein Rb in vulvar lesions of Chinese women. Gynecologic oncology. 1998;68(2):156–61. 10.1006/gyno.1997.4914 . 9514803

[23] Guerrero D, Guarch R, Ojer A, Casas JM, Mendez-Meca C, Esteller M, et al Differential hypermethylation of genes in vulvar cancer and lichen sclerosus coexisting or not with vulvar cancer. International journal of cancer. 2011;128(12):2853–64. 10.1002/ijc.25629 . 20734389

[24] Knopp S, Bjorge T, Nesland JM, Trope C, Scheistroen M, Holm R. p16INK4a and p21Waf1/Cip1 expression correlates with clinical outcome in vulvar carcinomas. Gynecologic oncology. 2004;95(1):37–45. 10.1016/j.ygyno.2004.07.026 . 15385108

[25] Lavorato-Rocha AM, Rodrigues IS, de Melo Maia B, Stiepcich MM, Baiocchi G, Carvalho KC, et al Cell cycle suppressor proteins are not related to HPV status or clinical outcome in patients with vulvar carcinoma. Tumour biology: the journal of the International Society for Oncodevelopmental Biology and Medicine. 2013;34(6):3713–20. 10.1007/s13277-013-0955-0 . 23832541

[26] Nogueira MC, Guedes Neto Ede P, Rosa MW, Zettler E, Zettler CG. Immunohistochemical expression of p16 and p53 in vulvar intraepithelial neoplasia and squamous cell carcinoma of the vulva. Pathology oncology research: POR. 2006;12(3):153–7. doi: Paor.2006.12.3.0153. . 16998595 10.1007/BF02893362

[27] van der Avoort IA, Shirango H, Hoevenaars BM, Grefte JM, de Hullu JA, de Wilde PC, et al Vulvar squamous cell carcinoma is a multifactorial disease following two separate and independent pathways. International journal of gynecological pathology: official journal of the International Society of Gynecological Pathologists. 2006;25(1):22–9. . 16306780 10.1097/01.pgp.0000177646.38266.6a

[28] Hao S. Expression and significance of p16 and Cyclin D1 in vulvar neoplasia.M.Sc. Thesis.China Medical University.2004. Available: http://www.cnki.net/KCMS/detail/detail.aspx?QueryID=6&CurRec=1&recid=&filename=2004139508.nh&dbname=CMFD0506&dbcode=CMFD&pr=&urlid=&yx=&v=MTc2NzMzcVRyV00xRnJDVVJMeWZaT2RtRnkvbVVyM0JWMTI3R3JLN0Y5VE1wNUViUElSOGVYMUx1eFlTN0RoMVQ.

[29] Ma H, Sun J. The Relationship between P16 expression and vulnerability and clinicopathological characteristics of vulvar prickle cell carcinoma. Chin J Clin Oncol Rehabil.2013; (01):6–8.

[30] Ma H (2002) Study of expression of PTEN and p16 gene in vulvar cancer. M.Sc. Thesis. Qingdao University. 2002. Available: http://www.cnki.net/KCMS/detail/detail.aspx?QueryID=15&CurRec=1&recid=&filename=2002093004.nh&dbname=CMFD9904&dbcode=CMFD&pr=&urlid=&yx=&v=MDc0NDExMjdITE94SGRITXE1RWJQSVI4ZVgxTHV4WVM3RGgxVDNxVHJXTTFGckNVUkx5ZlpPZG1GeS9tV3IzUFY=.

[31] Wei L, Pu D, Yin L. Expression and significance of p16 protein and PCNA in keratinizing vulva squamous cell carcinoma. Maternal and Child Health Care of China. 2008; (22):3168–3170.

[32] Zheng A, Peng Z, Wang H. Expression of P16 protein in premalignant lesion and caecinoma of vulvar.Sichuan Medical Journal. 2000; (05):405–406.

[33] Santos M, Landolfi S, Olivella A, Lloveras B, Klaustermeier J, Suarez H, et al p16 overexpression identifies HPV-positive vulvar squamous cell carcinomas. The American journal of surgical pathology. 2006;30(11):1347–56. 10.1097/01.pas.0000213251.82940.bf . 17063073

[34] Alonso I, Fuste V, del Pino M, Castillo P, Torne A, Fuste P, et al Does human papillomavirus infection imply a different prognosis in vulvar squamous cell carcinoma? Gynecologic oncology. 2011;122(3):509–14. 10.1016/j.ygyno.2011.05.016 . 21652058

[35] de Sanjose S, Alemany L, Ordi J, Tous S, Alejo M, Bigby SM, et al Worldwide human papillomavirus genotype attribution in over 2000 cases of intraepithelial and invasive lesions of the vulva. European journal of cancer (Oxford, England: 1990). 2013;49(16):3450–61. 10.1016/j.ejca.2013.06.033 . 23886586

[36] Missaoui N, Abdelkarim SB, Mokni M, Hmissa S. p16INK4A expression in squamous cell carcinomas of the vagina and the vulva in Tunisian women. Asian Pacific journal of cancer prevention: APJCP. 2014;15(24):10803–8. . 25605180 10.7314/apjcp.2014.15.24.10803

[37] Koh J, Enders GH, Dynlacht BD, Harlow E. Tumour-derived p16 alleles encoding proteins defective in cell-cycle inhibition. Nature. 1995;375(6531):506–10. 10.1038/375506a0 . 7777061

[38] Baylin SB, Herman JG. DNA hypermethylation in tumorigenesis: epigenetics joins genetics. Trends in genetics: TIG. 2000;16(4):168–74. Epub 2000/03/24. . 10729832 10.1016/s0168-9525(99)01971-x

[39] Bu J, Li H, Liu LH, Ouyang YR, Guo HB, Li XY, et al P16INK4a overexpression and survival in osteosarcoma patients: a meta analysis. International journal of clinical and experimental pathology. 2014;7(9):6091–6. ; PubMed Central PMCID: PMCPmc4203227. 25337256 PMC4203227

[40] Lin J, Albers AE, Qin J, Kaufmann AM. Prognostic significance of overexpressed p16INK4a in patients with cervical cancer: a meta-analysis. PloS one. 2014;9(9):e106384 10.1371/journal.pone.0106384 ; PubMed Central PMCID: PMCPmc4154680. 25188353 PMC4154680

[41] Horn LC, Reichert A, Oster A, Arndal SF, Trunk MJ, Ridder R, et al Immunostaining for p16INK4a used as a conjunctive tool improves interobserver agreement of the histologic diagnosis of cervical intraepithelial neoplasia. The American journal of surgical pathology. 2008;32(4):502–12. 10.1097/PAS.0b013e31815ac420 . 18223479

[42] Sznurkowski JJ, Milczek T, Emerich J. Prognostic factors and a value of 2009 FIGO staging system in vulvar cancer. Archives of gynecology and obstetrics. 2013;287(6):1211–8. 10.1007/s00404-012-2683-x ; PubMed Central PMCID: PMCPmc3655214. 23263173 PMC3655214

[43] Maggino T, Landoni F, Sartori E, Zola P, Gadducci A, Alessi C, et al Patterns of recurrence in patients with squamous cell carcinoma of the vulva. A multicenter CTF Study. Cancer. 2000;89(1):116–22. . 10897008 10.1002/1097-0142(20000701)89:1<116::aid-cncr16>3.0.co;2-4

[44] Burger MP, Hollema H, Emanuels AG, Krans M, Pras E, Bouma J. The importance of the groin node status for the survival of T1 and T2 vulval carcinoma patients. Gynecologic oncology. 1995;57(3):327–34. 10.1006/gyno.1995.1151 . 7774836

[45] Kosary CL. FIGO stage, histology, histologic grade, age and race as prognostic factors in determining survival for cancers of the female gynecological system: an analysis of 1973–87 SEER cases of cancers of the endometrium, cervix, ovary, vulva, and vagina. Seminars in surgical oncology. 1994;10(1):31–46. . 8115784 10.1002/ssu.2980100107

[46] Blecharz P, Karolewski K, Bieda T, Klimek M, Pudelek J, Kojs E, et al Prognostic factors in patients with carcinoma of the vulva—our own experience and literature review. European journal of gynaecological oncology. 2008;29(3):260–3. . 18592791

[47] Harada H, Nakagawa K, Iwata S, Saito M, Kumon Y, Sakaki S, et al Restoration of wild-type p16 down-regulates vascular endothelial growth factor expression and inhibits angiogenesis in human gliomas. Cancer research. 1999;59(15):3783–9. . 10446996

[48] Malesci A, Laghi L, Bianchi P, Delconte G, Randolph A, Torri V, et al Reduced likelihood of metastases in patients with microsatellite-unstable colorectal cancer. Clinical cancer research: an official journal of the American Association for Cancer Research. 2007;13(13):3831–9. 10.1158/1078-0432.ccr-07-0366 . 17606714

[49] Tada T, Watanabe T, Kazama S, Kanazawa T, Hata K, Komuro Y, et al Reduced p16 expression correlates with lymphatic invasion in colorectal cancers. Hepato-gastroenterology. 2003;50(54):1756–60. . 14696398

[50] Fakhry C, Westra WH, Li S, Cmelak A, Ridge JA, Pinto H, et al Improved survival of patients with human papillomavirus-positive head and neck squamous cell carcinoma in a prospective clinical trial. Journal of the National Cancer Institute. 2008;100(4):261–9. 10.1093/jnci/djn011 . 18270337

[51] Beutner KR, Tyring S. Human papillomavirus and human disease. The American journal of medicine. 1997;102(5a):9–15. . 9217657 10.1016/s0002-9343(97)00178-2

[52] Bloss JD, Liao SY, Wilczynski SP, Macri C, Walker J, Peake M, et al Clinical and histologic features of vulvar carcinomas analyzed for human papillomavirus status: evidence that squamous cell carcinoma of the vulva has more than one etiology. Human pathology. 1991;22(7):711–8. . 1649118 10.1016/0046-8177(91)90294-y

[53] Munger K, Howley PM. Human papillomavirus immortalization and transformation functions. Virus research. 2002;89(2):213–28. . 12445661 10.1016/s0168-1702(02)00190-9

[54] Zhao F, Chen Y, Wu Q, Wang Z, Lu J. Prognostic value of CD117 in cancer: a meta-analysis. International journal of clinical and experimental pathology. 2014;7(3):1012–21. ; PubMed Central PMCID: PMCPmc3971304. 24696718 PMC3971304

[55] Riethdorf S, Neffen EF, Cviko A, Loning T, Crum CP, Riethdorf L. p16INK4A expression as biomarker for HPV 16-related vulvar neoplasias. Human pathology. 2004;35(12):1477–83. . 15619206 10.1016/j.humpath.2004.09.004

[56] Bergeron C, Ordi J, Schmidt D, Trunk MJ, Keller T, Ridder R. Conjunctive p16INK4a testing significantly increases accuracy in diagnosing high-grade cervical intraepithelial neoplasia. American journal of clinical pathology. 2010;133(3):395–406. 10.1309/ajcpxsvcdz3d5mzm . 20154278

